# The impact of multiple gender dimensions on health-related quality of life in persons with Parkinson’s disease: an exploratory study

**DOI:** 10.1007/s00415-022-11228-2

**Published:** 2022-07-14

**Authors:** Irene Göttgens, Sirwan K. L. Darweesh, Bastiaan R. Bloem, Sabine Oertelt-Prigione

**Affiliations:** 1 Department of Primary and Community Care, Radboud Institute for Health Sciences, Radboud University Medical Center, Postbus 9101, 6500 HB Nijmegen, The Netherlands; 2 Department of Neurology, Center of Expertise for Parkinson and Movement Disorders, Donders Institute for Brain, Cognition and Behavior, Radboud University Medical Center, Nijmegen, The Netherlands

**Keywords:** Gender, Quality of Life, Parkinson’s disease, Methodology

## Abstract

**Background:**

There is a growing recognition that sex characteristics and gender-related aspects can have a substantial impact on the health-related quality of life (HRQoL) of persons with Parkinson’s disease (PD). Gender is a multidimensional construct, including dynamic social norms and relations that influence health and impact quality of life. Even when gender is investigated in the field of PD, it is frequently conceptualized as gender identity while other dimensions, such as roles or relations, are generally ignored. The aim of this study was to explore the impact of several gender dimensions on HRQoL among people with PD.

**Methods:**

We performed a survey-based, cross-sectional study in the Netherlands to explore the impact of several gender dimensions, namely; gender identity, gender roles and gender relations on HRQoL (PDQ-39) of people with PD.

**Results:**

In our study population (*N* = 307), including 127 (41%) women, we did not observe an association between gender identity and overall HRQoL. In contrast, an androgynous gender role and higher engagement in household tasks were associated with better overall HRQoL among people with PD.

**Conclusions:**

This study offers the first detailed description of the impact of different gender dimensions on the HRQoL of people with PD and highlights the need for more precise gender-measures to inform actionable gender-sensitive health interventions for people with PD.

**Supplementary Information:**

The online version contains supplementary material available at 10.1007/s00415-022-11228-2.

## Introduction

Parkinson’s disease (PD) is the second most common neurodegenerative disease worldwide and an increasing challenge to global health due to its rapidly rising prevalence [1, 2]. This trend places a considerable burden on societies, individuals and health systems, as PD-related disabilities significantly reduce health-related quality of life (HRQoL) [3]. In addition, the current knowledge base around PD poorly represents the diversity of people that live with the disease. The under-representation of different socioeconomic and ethnic groups, as well as women in PD research, result in an incomplete picture of the true impact on individual quality of life. [4–7]

There is a growing recognition of the need to increase diversity and representation in PD studies, especially a better consideration of sex and gender. Studies into sex-linked associations with PD have reported a higher risk in females of developing dyskinesia, and a lower risk of developing cognitive impairments compared to male patients [8, 9]. Nevertheless, the pathophysiological insights underlying such sex-specificity in determining PD-associated risks remain scarce. Furthermore, findings about the impact of gender on HRQoL among people with PD are inconclusive to date, highlighting the need for methodologically sound sex and gender sensitive clinical research [10–12]. Aside from potential differences in sex, which refer to a spectrum of biological and physiological characteristics, people with PD may also differ in gender, which refers to a multidimensional continuum of socially constructed behaviors, roles and relations associated with men, women and gender-diverse people [13]. The association between sex-linked characteristics, gender-related aspects and a given health outcome, can depend on one, both or neither of the two concepts. Therefore, any investigation within this field requires critical conceptual clarity in the operationalization of sex and gender [14].

Gender-related aspects are essential elements of people’s lived experiences and entail dynamic social norms and relations that influence health and quality of life [15]. Studies that investigate the impact of gender on PD have almost exclusively focused on self-reported gender identity and rarely included other dimensions of gender. Moreover, if self-reported gender identity is included in PD research, it is often applied as an all-encompassing representation of the construct “gender” and used interchangeably with the construct of “sex” or used as a proxy for biological sex-linked characteristics [10, 16, 17]. This lack of conceptual clarity limits the generalizability of these research findings and contributes to an incomplete representation of gender, its potential interaction with sex-linked characteristics and its impact on health of people with PD [14].

It is important to precisely study the impact of different gender dimensions in the context of PD, because social roles and relations can be affected by emerging disabilities and can change over time due to a growing burden of disease [18]. The objective of this study is to explore the impact of the distinct gender dimensions of gender identity, gender roles and gender relations, on the health-related quality of life in people with Parkinson’s disease.

## Methods

### Study design

We conducted a cross-sectional survey study among persons with PD living in the Netherlands. We recruited the participants between March 2020 and March 2021 as part of a large prospective cohort study; the PRIME Parkinson Evaluation Study (PRIME-NL Study) [19]. The PRIME-NL Study has been approved by the Ethical Board of the Radboud University Medical Center (CMO file number 2019–5618). All participants signed a digital informed consent before inclusion in the study.

### Study population

Participants were eligible for this study if they met the following criteria: Diagnosed with Parkinson’s disease or Parkinsonism; 18 years of age or older; Able to read and understand Dutch; Willing and able to complete an online survey; Providing digital written informed consent.

### Clinical assessments

The following demographic and clinical data were recorded: age, disease duration, years living with symptoms, clinical disease duration, and education level.

## Determinants

### Outcome measure

#### Health-related quality of life

We used the Parkinson Disease Questionnaire (PDQ-39), a disease-specific, a self-evaluative health-related quality of life (HRQoL) instrument, to assess HRQoL. Participants are asked to score each of the 39 items on a 5-point Likert scale from 0 (never) to 4 (always). Followingly, we calculated the eight subscale scores and an index summary score (PDQ-SI), with all answers being transformed to a 0–100 scale and higher scores representing worse HRQoL. The eight PDQ-39 subscales are mobility (MOB), activities of daily living (ADL), emotional well-being (EMO), stigma (STIG), social support (SOC), cognitive impairment (COG), communication (COM), and bodily discomfort (BOD).

#### Gender dimensions assessments

To capture multiple gender dimensions in our survey, we performed a literature review to identify state-of-the-art options for operationalizing the gender dimensions applied to this study: Gender identity, gender roles and gender relations (Supplement 1).

#### Gender identity

The dimension of gender identity refers the gendered sense of self of a person [20]. Gender identity was operationalized through self-reported gender identity, sex assigned at birth and sexual orientation. Self-reported gender identity refers to the self-identification of a person—the response options were woman/man/non-binary or a ‘none of the above’ with an open text option. Sex assigned at birth refers to the sex categorization of a person at birth, with the response options of female/male/intersex/other. Sexual orientation refers to the gender identity of those to whom a person is sexually and romantically attracted to, with response options of heterosexual/homosexual/bisexual/other.

#### Gender roles

Gender roles refers to stereotypical behaviors, roles and attitudes that are defined, in a specific cultural context, as more appropriate or desirable for men or women. Gender roles was operationalized through gender expression and gender role orientation. Gender expression refers to how feminine or masculine people see and present themselves. We measured gender expression with a unidimensional 7-point Likert scale, ranging from very feminine to very masculine [20]. Gender role orientation refers a person’s orientation towards personality traits that are culturally associated with stereotypical masculine and feminine behaviors. The 60-item Bem Sex Role Inventory (BSRI) was used to assess people’s perceptions of their psychological gender role orientation. [21] The BSRI measures stereotypical masculine and feminine personality traits as independent dimensions, thereby making it possible to characterize a person as masculine, feminine, androgynous or undifferentiated as a function of the difference between their endorsement of masculine and feminine characteristics. The instrument uses a 7-point Likert scale ranging from 1 (never or almost never true) to 7 (always or almost always true) for stereotypically masculine (*n* = 20; e.g., ambitious, dominant) and feminine (*n* = 20; e.g., affectionate, gentle) descriptors, plus neutral filler items (*n* = 20; e.g., sincere, conscientious). Individuals with an androgynous self-concept score high on both the masculine and feminine characteristics, while the undifferentiated individuals score low on both masculine and feminine characteristics. People with a strong masculine or feminine self-concept score high only one of these dimensions.

#### Gender relations

Gender relations define how people, according to cultural context, interact with others and how others relate to them, depending on their attributed sex or perceived gender identity [22]. Gender relational experiences occur on personal and intimate levels as well as on societal and institutional levels.[23]. For the purpose of this study, we focused on gender relations in the private domain and the medical domain.

We operationalized gender relations in the private domain through living situation and childcare, division of household labor, relative household income, paid and unpaid labor. Living situation was assessed by asking participants about their marital/partner status (living with/without a partner/spouse) and whether they were taking care of children (With/without children living at home).

Division of household labor was measured with the question: “In your household, who usually does the following task?”. Participant rated their housework responsibilities on 7 core tasks (cooking meals, cleaning the kitchen, grocery shopping, house cleaning, laundry, maintenance and repairs and financial administration) [24–26]. Response options were: spouse/partner, shared equally, respondent, or someone else. Mean scores were computed, with higher scores indicating increased participant involvement in housework. Division of household tasks was recoded as unequally distributed or equally distributed between spouses/partners in their household.

Relative household income was assessed by asking participants about their proportional earnings in their household. Relative income was categorized ranging from 0 to 100%. Relative income was recoded and labeled unequally distributed or equally distributed between spouses/partners in their household.

Paid and unpaid labor was measured with the question: “On average, how many hours a week do you usually do paid/unpaid work?” and categorized into 4 categories for paid and unpaid work.

Gender relations in the medical domain was operationalized though the attributed gender identity of the primary and attending healthcare provider by the participant. The primary health care provider was defined as “the PD related healthcare provider that the participant visits most often” and the attending healthcare provider was defined as “the PD related healthcare provider who is considered the main responsible care provider by the participant”.

### Pre-testing of the gender assessments

The survey was pre-tested in a convenience sample of 10 random patients, diverse in age and gender identity. The survey pre-test was performed digitally with regards to comprehension, answer retrieval, comfortability with answering the questions and completeness of the response options per item. The survey was optimized based on the pre-test feedback. Tourangeau's four-stage model was used to inspire the development of the pre-test evaluation questions [27, 28].

### COVID-19 stressors questionnaire

Since April 2020, the PRIME-NL questionnaire included eight statements about different situations that could have occurred during the COVID-19 pandemic, based on the DynaCORE questionnaire [29]. The question that accompanied each statement was: ‘Could you indicate how you experience or experienced these situations because of the COVID-19 pandemic?’ Each question was scored on a six-point Likert-scale ranging from ‘this situation did not occur’ to ‘very troublesome’. A social stressors score was calculated, summarizing statements about loss of social contacts, cancellation of social events and tension or conflict at home, and a care stressors score, summarizing statements about problems with access to care, medication and nursing. Two additional COVID-19 stressors, regarding COVID-19 symptoms and physical activity and relaxation, were not included in the sub scores, but were summed up in the stressors sum score including all eight items. A detailed description of the questionnaire can be found in Supplement A.

### Statistical analysis

We performed descriptive statistics on the participants demographic and gender dimension variables. Differences between demographic, gender related data with sex assigned at birth and gender identity were compared using Kruskal–Wallis rank sum test or the Fisher exact test.

For univariate and multivariate regression analyses of gender dimensions and HRQoL, self-reported gender identity was included as a proxy for the dimension of gender identity, gender role orientation (BSRI) for the dimension of gender roles and household task division and relative income for the dimension of gender relations. Living situation included a dichotomous measure of being married/living with a partner or not and was used as a determinant for private gender relations. Therefore, only participants that indicated that they were married/living together with a partner were included in the analyses related to gender relations.

The association between (1) gender identity (self-reported gender identity) and HRQoL (PDQ-SI scores); (2) gender role orientation (BSRI score) and HRQoL; and 3) gender relations (household task division and relative income) and HRQoL was determined using multiple linear regression, which were adjusted for age, clinical disease duration and COVID-19 stressors. A multiplicity adjusted *P* value < 0.0127 indicated statistical significance for the PDQ-SI scores. Statistical analyses were performed using R Studio Version 1.1.463. The data that support the findings of this study are available from the corresponding author upon reasonable request.

## Results

### Population characteristics

A total of 307 people with PD were included, of which 127 (41%) were female and 179 (58%) were male and 1 (0.6%) person was self-reported as intersex (Supplement 2). The mean age was 67.5 ± 8.3 years and the mean age at diagnosis was 61.5 ± 9.4 years. These subgroup characteristics correspond with the baseline characteristics of the PRIME cohort [30]. Differences in clinical characteristics were observed between the sexes with females with PD being younger in both current age (*p* = 0.002) and age at diagnosis (*p* < 0.001), they had a longer disease duration (*p* = 0.009) and had relatively more comorbidities from musculoskeletal diseases compared to the males in our sample (*p* = 0.006). In contrast, no significant differences were found between the reported sex assigned at birth and education level, Self-Assessement Parkinson’s Disease Disability Scale score (SPDDS), Parkinson Disease Questionnaire Summary Index score (PDQ-39 SI) and COVID-19 stressor score.

On the dimension of Gender Identity, 127 (41%) as woman and 180 (59%) participants identified as man. None of the participants identified as non-binary or otherwise and 96% of the participants were heterosexual (Table 1). Significant differences in gender characteristics were found between gender identities (*p* < 0.001) with women being less represented than men in our sample.

**Table 1:** Characteristics related to gender dimensions of the study population

	Overall (*N* = 307) *N* (%)	Women (*N* = 127) *N* (%)	Men (*N* = 180) *N* (%)
*Gender identity*			
Sex assigned at birth			
Female	127 (41)	127 (100)	0 (0)
Intersex	1 (0.3)	0 (0)	1 (0.6)
Male	179 (58)	0 (0)	179 (99)
Self-reported gender identity			
Woman	127 (41)	127 (100)	0 (0)
Man	180 (59)	0 (0)	180 (100)
Non-binary	0 (0)	0 (0)	0 (0)
None of the above	0 (0)	0 (0)	0 (0)
Sexual orientation			
Heterosexual	291 (96)	119 (95)	172 (96)
Bisexual	7 (2.3)	2 (1.6)	5 (2.8)
Homosexual	6 (2.0)	4 (3.2)	2 (1.1)
Unknown	3	2	1
*Gender roles*			
Gender expression			
Feminine	112 (36)	112 (88)	0 (0)
Both masculine and feminine	31 (10)	15 (12)	16 (8.9)
Masculine	164 (53)	0 (0)	164 (91)
Gender role orientation			
Feminine	97 (32)	64 (51)	33 (18)
Androgynous	46 (15)	14 (11)	32 (18)
Masculine	58 (19)	9 (7.1)	49 (27)
Undifferentiated	106 (34)	40 (31)	66 (37)
*Gender relations*			
Living situation			
Married/With partner	241 (81)	87 (72)	154 (88)
Not married/Without partner	55 (19)	34 (28)	21 (12)
Unknown	11	6	5
Childcare			
With children living at home	33 (11)	16 (13)	17 (9.8)
Without children living at home	263 (89)	105 (87)	158 (90)
Unknown	11	6	5
Division of household labor			
Household labor score (Mean ± SD)*	12.8 ± 3.2	14.1 ± 3.8	12.1 ± 2.6
Equally distributed	115 (48)	39 (46)	76 (49)
Unequally distributed	123 (52)	45 (54)	78 (51)
Unknown	3	3	0
Relative income			
0–25%	26 (12)	24 (35)	2 (1)
26–0%	46 (21)	21 (30)	25 (17)
51–75%	67 (31)	20 (29)	47 (32)
76–100%	77 (36)	4 (6)	73 (50)
Equally distributed	113 (52)	41 (59)	72 (49)
Unequally distributed	103 (48)	28 (41)	75 (51)
Unknown	25	18	7
Paid work			
None	251 (82)	107 (86)	144 (80)
1–20 h	26 (8.5)	12 (9.6)	14 (7.8)
21–40 h	23 (7.5)	5 (4.0)	18 (10)
More than 40 h	5 (1.6)	1 (0.8)	4 (2.2)
Unknown	2	2	0
Unpaid work			
None	72 (24)	27 (22)	45 (26)
1–10 h	169 (57)	67 (54)	102 (59)
11–20 h	44 (15)	23 (18)	21 (12)
More than 20 h	14 (4.7)	8 (6.4)	6 (3.4)
Unknown	8	2	6

On the dimension of Gender Roles, 112 women (88%) scored themselves as mostly or strongly feminine, whereas 164 men (91%) scored themselves mostly or strongly masculine on the unidimensional gender expression scale. However, the gender role orientation (BSRI) score showed that 106 participants (35%) scored low on both masculine and feminine personality traits and 97 (32%) scored high on only feminine traits. Forty-six participants (15%) were classified androgynous, scoring high on both masculine and feminine traits. Significant differences were observed between the unidimensional measure of masculine and feminine gender expression and the two-dimensional gender role orientation scale measured by the BSRI (*p* < 0.001).

On the dimension of Gender Relations in the private domain, 239 (81%) participants indicated to be married or to live together with a partner and the majority (89%) reported no children living at home. For the group that was married and/or lived together with a partner, the household task division was equally distributed in 48% of the cases, whereas relative income was equally distributed in 52% of the cases. The majority of the participants did not perform any paid work (82%) and performed between 1 and 10 h of unpaid work (57%) on average on a weekly basis. Significant differences were found between household task divisions and relative income and gender identity, with men being less engaged with household task (*p* < 0.001) and having more relative income (*p* < 0.001) compared to their partner/spouse.

On the dimension of gender relations in the medical domain, participants indicated that their primary healthcare provider (defined as “the PD related healthcare provider that the participant visits most often”) was in most cases the physiotherapist (55%), followed by the neurologist (21%) (Supplement 3). The attending healthcare provider (defined as “the PD related healthcare provider who is considered the main responsible care provider by the participant”) was in the majority of the cases the neurologist (87%), followed by the general practitioners in 9% of the cases. Significant differences were found between the gender identity of the participants and the reported gender identity of their treating neurologist, with women with PD (76/127 (61%)) visiting a female neurologist more often than men (78/180 (44%)) (*p* = 0.004).

### Associations between gender dimensions in the private domain and health-related quality of life

Self-reported gender identity did not show a significant association with overall HRQoL (PDQ-39 index score) (Table 2). In contrast, the results of the Bem Sex Role Inventory showed that an androgynous gender role significantly predicted a better overall HRQoL (*B* = − 5.55, *p* = 0.009), compared to all the other gender roles. Backwards regression showed that specifically the gender-related traits of “Athletic”, “Assertive”, “Self-sufficient” and “Happy” were contributing to better overall HRQoL. The results on the dimension of gender relations showed that higher engagement with household tasks was associated with slightly better overall HRQoL (B-0.86, *p* = 0.002). No significant association was found between equal distribution of household tasks and HRQoL. Furthermore, a nominally significant association was found between equal distribution of relative income and better overall HRQoL (*B* = − 3.55, *p* = 0.048).

**Table 2:** Interaction between gender identity, gender role and gender relations in the private domain and health-related quality of life

Gender Identity	Health-related quality of life (PDQ-39)								
	PDQ-SI	MOB	ADL	EMO	STIG	SOC	COG	COM	BOD
	*ß* (SE)	*ß* (SE)	*ß* (SE)	*ß* (SE)	*ß* (SE)	*ß* (SE)	*ß* (SE)	*ß* (SE)	*ß* (SE)
Self-reported gender identity									
Woman	1.67 (1.60)	9.89*** (2.79)	0.71 (2.55)	4.04 (2.18)	2.34 (2.28)	3.15 (2.29)	− 5.15* (2.12)	− 8.05*** (2.39)	6.39* (2.56)
*Gender roles*									
Gender role orientation									
Feminine	2.85 (1.67)	5.14 (2.98)	1.93 (2.66)	4.26 (2.28)	2.31 (2.39)	2.08 (2.40)	2.84 (2.24)	− 1.09 (2.56)	5.30 (2.69)
Androgynous	− 5.55^2^ (2.12)	− 8.35* (3.81)	− 7.48* (3.38)	− 3.33 (2.94)	− 6.79* (3.04)	− 5.03 (3.07)	− 0.68 (2.88)	− 6.34 (3.26)	− 6.38 (3.45)
Masculine	− 2.45 (1.90)	− 5.25 (3.39)	− 2.34 (3.03)	− 6.06* (2.58)	− 1.01 (2.72)	− 1.01 (2.73)	0.36 (2.55)	− 0.09 (2.91)	− 4.18 (3.07)
Undifferentiated	2.36 (1.65)	3.90 (2.94)	4.30 (2.61)	2.40 (2.26)	2.53 (2.35)	1.71 (2.37)	− 2.63 (2.21)	4.87 (2.51)	1.77 (2.67)
*Gender relations*									
Household task division									
Household labor score	− 0.86^2^ (0.28)	− 1.87*** (0.49)	− 1.57*** (0.44)	− 0.53 (0.38)	− 0.05 (0.37)	0.03 (0.38)	− 0.96** (0.37)	-1.28** (0.42)	− 0.62 (0.45)
Equally distributed	− 2.37 (1.76)	− 5.61 (3.11)	− 5.85* (2.77)	− 3.87 (2.34)	− 1.96 (2.29)	− 0.77 (2.39)	0.54 (2.31)	− 0.85 (2.67)	− 0.62 (2.81)
Relative income									
0–25%	− 1.04 (3.37)	6.71 (5.92)	− 6.23 (5.10)	3.89 (4.67)	− 4.47 (4.16)	− 3.32 (4.92)	− 0.85 (4.54)	− 6.15 (5.06)	2.08 (5.54)
26–50%	− 2.23 (2.49)	− 2.24 (4.37)	− 6.03 (3.76)	− 3.35 (3.44)	− 3.02 (3.07)	− 2.15 (3.63)	− 0.17 (3.35)	− 3.54 (3.73)	2.69 (4.09)
51–75%	− 3.50 (2.17)	− 1.07 (3.80)	− 5.73 (3.28)	− 4.98 (3.00)	− 2.22 (2.67)	− 2.94 (3.16)	− 1.23 (2.92)	− 5.87 (3.25)	− 3.94 (3.56)
Equally distributed	− 3.55^1^ (1.78)	− 4.61 (3.20)	− 5.31 (2.70)	− 5.34* (2.43)	− 2.73 (2.25)	− 2.36 (2.55)	− 1.27 (2.37)	− 5.27 (2.71)	− 1.47 (2.93)

PDQ Index Score: ^1^*p* = [0.0127 – 0.050]; ^2^*p* < 0.0127

PDQ Single Domain Scores: *p < 0.05; **p < 0.01; ***p < 0.001

Gender identity category ‘non-binary’ is excluded from the table due to the absence of results in this category

For each independent variable, the analysis was adjusted for age and disease duration and COVID-19 stressor sum score. *ß* coefficients are presented for gender roles as compared to the other categories (category (1)—reference groups (0)). For relative income, the 75–100% category was used as reference group

### Associations between gender relations in the medical domain and health-related quality of life

No significant differences were found between the reported gender identity of the primary or attending healthcare provider and overall HRQoL of the participants (Table 3).

**Table 3:** Interactions between gender relations in the medical domain and health-related quality of life

	Health-related quality of life (PDQ-39)								
	PDQ-SI	MOB	ADL	EMO	STIG	SOC	COG	COM	BOD
	ß (SE)	ß (SE)	ß (SE)	ß (SE)	ß (SE)	ß (SE)	ß (SE)	ß (SE)	ß (SE)
Primary healthcare provider									
Same gender identity	1.66 (1.50)	1.83 (2.70)	− 0.01 (2.38)	4.05* (2.00)	0.11 (2.07)	4.76* (2.00)	− 0.40 (1.93)	1.37 (2.32)	1.56 (2.42)
Women provider	− 2.81 (1.56)	− 0.62 (2.81)	− 2.52 (2.50)	− 2.88 (2.11)	− 1.81 (2.17)	− 4.02 (2.10)	− 4.12* (2.00)	− 3.02 (2.42)	− 3.47 (2.53)
Attending healthcare provider									
Same gender identity	2.22 (1.66)	4.10 (3.00)	2.51 (2.66)	1.64 (2.23)	2.90 (2.26)	2.29 (2.31)	− 0.09 (2.10)	3.22 (2.54)	1.21 (2.67)
Women provider	− 1.86 (1.65)	1.46 (2.98)	− 3.76 (2.63)	0.84 (2.21)	− 0.61 (2.24)	− 2.68 (2.28)	− 3.51 (2.06)	− 6.31* (2.49)	− 0.28 (2.65)

PDQ Index Score: ^1^*p* = [0.0127 – 0.050]; ^2^*p* < 0.0127

PDQ Single Domain Scores: *p < 0.05; **p < 0.01; ***p < 0.001

*ß* coefficients are presented for categorical variables as compared to the other category (category (1)—reference group (0))

## Discussion

We conducted the present study to explore the impact of the different gender dimensions—gender identity, gender role orientation and gender relations—on health-related quality of life (HRQoL) among people with Parkinson’s disease (PD). We found no significant association between self-reported gender identity and overall HRQoL, whereas an androgynous gender role orientation and higher engagement in household tasks (gender relations in the private domain) were each associated with better overall HRQoL among people with PD. These results highlight the need to specifically define and operationalize the gender dimensions under investigation to aid the clinical implementation of gender-sensitive results in the care of people with PD.

The impact of gender on clinical outcomes has been postulated in other fields [31, 32]; however, the use of composite indices rather than the investigation of specific gender dimensions limits the transferability of these findings into clinical practice. We recently demonstrated the impact of gender roles on HRQoL in long-term cancer survivors and their relative underestimation in men with cancer [33]. These results were only possible when disentangling the gender identity dimension from gender roles [34]. Although most current research focuses on gender identity, other dimensions such as gender roles, norms and behaviours probably impact health behaviour and illness more significantly. Our present study supports this assumption and offers the first detailed description of the impact of different gender dimensions on the QoL of people with PD. In fact, in our population gender identity did not impact overall HRQoL, yet an androgynous gender role orientation (GRO) associated with better overall HRQoL. This particular finding is in line with previous reports outlining the importance of gender roles in PD [35–37].

Overall, these findings build on the Sex Role Adaptability hypothesis stating that psychologically androgynous individuals are more flexible in their choice of situationally effective behaviors and can, hence, better adapt to varying challenges [38]. Psychosocial and behavioral interventions hold great promise as non-pharmacological approaches for managing a variety of motor and non-motor symptoms in PD, particularly in reducing stress, anxiety and depression; all of which impact HRQoL [39, 40]. Psychosocial interventions aiming to improve HRQoL of people with PD could strengthen a persons’ practice of supportive gender-related traits to cope with the evolving reality of a chronic disease and its impact on quality of life, while remaining attentive to their sociocultural normative aspects.

Previous literature described the impact of chronic disease, and PD in particular, on identity, loss of valued social roles and the development of new ones [41, 42]. In living with PD, the activities that define one’s identity and social relations decline as the disease progresses, leading to a potential loss of the former gender role [41]. These role changes can have an overwhelming negative impact on psychological well-being and quality of life [43]. In line with this, the behaviors attributed to impulse control disorders related to dopamine replacement therapy in PD could be seen as an attempt to embolden one´s gender role. Previous studies have reported a higher prevalence of impulse control behaviors (ICB), such as hypersexuality and gambling behaviors in men, while compulsive buying appears more common among women with PD [44, 45]. It remains to be investigated whether these differences in behavioral expressions related to ICB are due to differences in pathophysiology or a result of socially acceptable gender-related behaviors that reinforce gender roles.

In our study population, more engagement in household tasks associated with slightly better overall health-related quality of life. More engagement might be explained by less PD-related disabilities and, therefore, higher HRQoL. However, unequal gender relations in household labor negatively affected the HRQoL of women more compared to men, potentially due to traditional gender relations that attribute the burden of household and informal care work mostly to women regardless of mounting PD-related disabilities [46]. In line with earlier findings [47], our study suggests that relatively equal financial resources in the relationship of people with PD and their partner, slightly improved their health-related quality of life, possibly due to reduced financial stressors. Equal relative income distribution between partners/spouses could also potentially reduce financial stressors due to less dependency on a single income, which might be compromised if the person with PD is the primary provider.

### Study limitations

Gender assessments can contain sensitive questions and health researchers need to be mindful of the risk of social desirability bias. For this study, we strived to reduce socially desirable responses and non-responses using a validated questionnaire when available [21] and by systematically pre-testing survey items for which no validated or translated measure was available. In addition, we studied the effect of private gender relations through involvement in household tasks and relative income using independent samples of men and women, without collecting data from participant spouses/partners. We analyzed the perceptions of participants about their own involvement compared to their partner’s involvement. We also used the BSRI to operationalize gender roles. Although the BSRI has encountered criticism over the years [48], it is still the most widely used instrument to measure gender roles in healthcare. Nevertheless, the ongoing debate about the categorization of the investigated traits to feminine, masculine or androgynous can be problematic as described by Nielsen and colleagues [36]. For example, in our study we found that the gender-related traits of “Athletic”, “Assertive”, “Self-sufficient” and “Happy” were contributors to better overall HRQoL and these could possibly be used as direct predictors of HRQoL rather than as components of a specific gender role.

## Conclusions

This study offers a first detailed description of the impact of different gender dimensions on the QoL of people with PD. Our findings showed that specific gender dimensions can impact health-related quality of life differently among people with PD. Insights from this study help to improve gender-sensitive investigations by highlighting the need for more rigorous analysis regarding the impact of various gender dimensions on the quality of life and experience of care of people with PD. Particularly, more in-depth explorations into the significance of gender roles and relations on health behaviour can support clinicians in their considerations for more targeted gender-sensitive psychosocial interventions, which can contribute to important improvements in quality of life. Overall, the precise investigation of the impact of gender dimensions on PD holds much promise for targeted psychosocial interventions and should be further explored.

## Supplementary Information

Below is the link to the electronic supplementary material.

Supplementary file1 (DOCX 16 kb)

Supplementary file2 (DOCX 18 kb)

Supplementary file3 (DOCX 18 kb)

Supplementary file4 (DOCX 16 kb)
