# Supplementary material for: The impact of multiple gender dimensions on health-related quality of life in persons with Parkinson’s disease: an exploratory study
Source: J Neurol. 2022 Jul 14;269(11):5963–72. doi: 10.1007/s00415-022-11228-2 (PMC9281291; doi:10.1007/s00415-022-11228-2)
Supplement: Supplementary file 1 — Supplementary file1 (DOCX 16 kb) [file 415_2022_11228_MOESM1_ESM.docx]

**Supplement 1.** Overview of the included gender dimensions assessments

| **Dimension** | **Endpoint** | **Instrument** |
| --- | --- | --- |
| Gender identity | Self-defined gender identity | Single Item Question |
|  | Sex assigned at birth | Single Item Question |
|  | Sexual orientation | Single Item Question |
| Gender Roles | Gender Expression | Single Item Question |
|  | Gender Role Orientation | Bem Sex Role Inventory |
| Gender Relations | Private - Living Situation | Singe Item Question |
|  | Private - Childcare | Single Item Question |
|  | Private - Division of household labor | Seven Item Question |
|  | Private - Relative income | Single Item Question |
|  | Private – Paid and Unpaid labor | Single Item Question |
|  | Medical – Gender identity of the primary healthcare provider | Single Item Question |
|  | Medical – Gender identity of the attending healthcare provider | Single Item Question |

**Author Information:**

Irene Göttgens^1^*, Sirwan K.L. Darweesh^2^, Bastiaan R. Bloem^2^, Sabine Oertelt-Prigione^1^*.

^1^ Department of Primary and Community Care, Radboud Institute for Health Sciences, Radboud University Medical Center, Nijmegen, The Netherlands.

^2^ Department of Neurology, Center of Expertise for Parkinson & Movement Disorders, Donders Institute for Brain, Cognition and Behavior, Radboud University Medical Center, Nijmegen, The Netherlands.

*Corresponding Author

Irene Göttgens

Radboud University Medical Center

Department of Primary and Community Care

Postbus 9101, 6500 HB Nijmegen

The Netherlands

Email: Irene.gottgens@radboudumc.nl
