# Supplementary material for: The impact of multiple gender dimensions on health-related quality of life in persons with Parkinson’s disease: an exploratory study
Source: J Neurol. 2022 Jul 14;269(11):5963–72. doi: 10.1007/s00415-022-11228-2 (PMC9281291; doi:10.1007/s00415-022-11228-2)
Supplement: Supplementary file 2 — Supplementary file2 (DOCX 18 kb) [file 415_2022_11228_MOESM2_ESM.docx]

**Supplement 2.** General and clinical characteristics of the study population

|  | **Overall** (n= 307) Mean ± SD | **Female** (n= 127)  Mean ± SD | **Intersex** (n= 1)  Mean ± SD | **Male** (n= 179)  Mean ± SD |
| --- | --- | --- | --- | --- |
| Age (years) | 67.5 (8.3) | 65.6 (8.6) | 66.0 (NA) | 68.8 (7.8) |
| Age at diagnosis (years) | 61.5 (9.4) | 58.8 (9.7) | 60.0 (NA) | 63.5 (8.6) |
| Disease duration (year) | 6.1 (4.6) | 6.9 (4.7) | 6.0 (NA) | 5.5 (4.4) |
| SPDDS score (0 – 100) | 34.3 (10.9) | 36.0 (13.2) | 25.0 (NA) | 33.1 (8.7) |
| PDQ-39 SI Score (0 – 100) | 24.8 (12.5) | 26.5 (12.3) | 17.9 (NA) | 23.7 (12.6) |
| COVID-19 Stressor score (0-40) | 2.5 (0.9) | 2.5 (0.9) | 2.4 (NA) | 2.5 (0.9) |
|  | **Overall** (n= 307)  N (%) | **Female** (n= 127)  N (%) | **Intersex** (n= 1)  N (%) | **Male** (n= 179)  N (%) |
| Medication use for PD (Yes) | 294 (94) | 121 (96) | 1 (100) | 172 (97) |
| Education level |  |  |  |  |
| None | 0 (0) | 0 (0) | 0 (0) | 0 (0) |
| Primary education | 6 (2.0) | 2 (1.6) | 0 (0) | 4 (2.2) |
| Secondary – prevocational | 50 (16) | 25 (20%) | 0 (0) | 25 (14) |
| Secondary – higher | 30 (9.8) | 15 (12) | 0 (0) | 15 (8.4) |
| Intermediate – vocational | 54 (18) | 22 (17) | 0 (0) | 32 (18) |
| Higher - professional | 164 (54) | 62 (49) | 1 (100) | 101 (57) |
| Other | 1 (0.3) | 0 (0) | 0 (0) | 1 (0.6) |
| Comorbidities |  |  |  |  |
| Heart diseases | 62 (20) | 21 (17) | 0 (0) | 41 (23) |
| Lung diseases | 29 (9.5) | 11 (8.7) | 0 (0) | 18 (10) |
| Musculoskeletal diseases | 93 | 50 (40) | 0 (0) | 43 (24) |
| Neuropsychiatric diseases | 20 (6.6) | 8 (6.3) | 0 (0) | 12 (6.7) |
| Endocrine or Metabolic diseases | 32 (10) | 13 (10) | 0 (0) | 19 (11) |
| Cancer | 16 (5.2) | 6 (4.8) | 1 (100) | 9 (5.1) |
| None of the above | 130 (43) | 52 (41) | 0 (0) | 78 (44) |

**Author Information:**

Irene Göttgens^1^*, Sirwan K.L. Darweesh^2^, Bastiaan R. Bloem^2^, Sabine Oertelt-Prigione^1^*.

^1^ Department of Primary and Community Care, Radboud Institute for Health Sciences, Radboud University Medical Center, Nijmegen, The Netherlands.

^2^ Department of Neurology, Center of Expertise for Parkinson & Movement Disorders, Donders Institute for Brain, Cognition and Behavior, Radboud University Medical Center, Nijmegen, The Netherlands.

*Corresponding Author

Irene Göttgens

Radboud University Medical Center

Department of Primary and Community Care

Postbus 9101, 6500 HB Nijmegen

The Netherlands

Email: Irene.gottgens@radboudumc.nl
