# Supplementary material for: The impact of multiple gender dimensions on health-related quality of life in persons with Parkinson’s disease: an exploratory study
Source: J Neurol. 2022 Jul 14;269(11):5963–72. doi: 10.1007/s00415-022-11228-2 (PMC9281291; doi:10.1007/s00415-022-11228-2)
Supplement: Supplementary file 3 — Supplementary file3 (DOCX 18 kb) [file 415_2022_11228_MOESM3_ESM.docx]

**Supplement 3.** Characteristics related to gender relations in the medical domain between study participants and healthcare providers.

|  | **Overall** (n= 307)  N (%) | **Women** (n= 127)  N (%) | **Men** (n= 180)  N (%) |
| --- | --- | --- | --- |
| **Healthcare Professionals’ Gender** |  |  |  |
| General Practitioner |  |  |  |
| Woman | 149 (50) | 65 (53) | 84 (49) |
| Man | 147 (50) | 58 (47) | 89 (51) |
| Unknown | 11 | 4 | 7 |
| Neurologist |  |  |  |
| Woman | 154 (51) | 76 (61) | 78 (44) |
| Man | 146 (49) | 48 (39) | 98 (56) |
| Unknown | 7 | 3 | 4 |
| Parkinson Nurse |  |  |  |
| Woman | 247 (96) | 98 (95) | 149 (97) |
| Man | 9 (3.5) | 5 (4.9) | 4 (2.6) |
| Unknown | 51 | 24 | 27 |
| Physiotherapist |  |  |  |
| Woman | 170 (67) | 34 (32) | 49 (34) |
| Man | 83 (33) | 73 (68) | 97 (66) |
| Unknown | 54 | 20 | 34 |
| Speech therapist |  |  |  |
| Woman | 98 (94) | 31 (100) | 67 (92) |
| Man | 6 (5.8) | 0 (0) | 6 (8.2) |
| Unknown | 203 | 96 | 107 |
| **Primary Healthcare Provider** |  |  |  |
| General Practitioner | 26 (9.4) | 10 (9) | 16 (9.6) |
| Neurologist | 57 (21) | 22 (20) | 35 (21) |
| Parkinson Nurse | 34 (12) | 9 (8.1) | 25 (15) |
| Physiotherapist | 154 (55) | 67 (60) | 87 (52) |
| Speech therapist | 7 (2.5) | 3 (2.7) | 4 (2.4) |
| Unknown | 29 | 16 | 13 |
| **Attending Healthcare Provider** |  |  |  |
| General Practitioner | 18 (7.6) | 7 (6.7) | 11 (8.3) |
| Neurologist | 206 (87) | 90 (86) | 116 (87) |
| Parkinson Nurse | 14 (5.9) | 8 (7.6) | 6 (4.5) |
| Physiotherapist | 0 (0) | 0 (0) | 0 (0) |
| Speech therapist | 0 (0) | 0 (0) | 0 (0) |
| Unknown | 69 | 22 | 47 |

**Author Information:**

Irene Göttgens^1^*, Sirwan K.L. Darweesh^2^, Bastiaan R. Bloem^2^, Sabine Oertelt-Prigione^1^*.

^1^ Department of Primary and Community Care, Radboud Institute for Health Sciences, Radboud University Medical Center, Nijmegen, The Netherlands.

^2^ Department of Neurology, Center of Expertise for Parkinson & Movement Disorders, Donders Institute for Brain, Cognition and Behavior, Radboud University Medical Center, Nijmegen, The Netherlands.

*Corresponding Author

Irene Göttgens

Radboud University Medical Center

Department of Primary and Community Care

Postbus 9101, 6500 HB Nijmegen

The Netherlands

Email: Irene.gottgens@radboudumc.nl
