# Supplementary material for: The impact of multiple gender dimensions on health-related quality of life in persons with Parkinson’s disease: an exploratory study
Source: J Neurol. 2022 Jul 14;269(11):5963–72. doi: 10.1007/s00415-022-11228-2 (PMC9281291; doi:10.1007/s00415-022-11228-2)
Supplement: Supplementary file 4 — Supplementary file4 (DOCX 16 kb) [file 415_2022_11228_MOESM4_ESM.docx]

**Supplement A.** Overview of COVID-19 Stressors.

| **Categorisation of stressors** | | **Stressors** | **Translated statements questionnaire** |
| --- | --- | --- | --- |
| COVID-19 stressor sum score | Care stressors | Problems access care | Problems with access to care |
|  |  | Problems access mediation | Problems with access to medication |
|  |  | Problems access nursing | Problems with access to nursing |
|  | Social stressors | Loss social contacts | Loss of social contacts |
|  |  | Social events cancelled | Social events which are cancelled |
|  |  | Tension or conflict at home | Tension or conflict at home |
|  |  | Unable to perform physical activity or to relax | Not being able to perform physical activity or to relax |
|  |  | COVID-19 symptoms | Showing COVID-19 symptoms or symptoms that could be related to COVID-19 |

Question that accompanied the statements: ‘Could you indicate how you experience or experienced these situations because of the COVID-19 pandemic?’

**Author Information:**

Irene Göttgens^1^*, Sirwan K.L. Darweesh^2^, Bastiaan R. Bloem^2^, Sabine Oertelt-Prigione^1^*.

^1^ Department of Primary and Community Care, Radboud Institute for Health Sciences, Radboud University Medical Center, Nijmegen, The Netherlands.

^2^ Department of Neurology, Center of Expertise for Parkinson & Movement Disorders, Donders Institute for Brain, Cognition and Behavior, Radboud University Medical Center, Nijmegen, The Netherlands.

*Corresponding Author

Irene Göttgens

Radboud University Medical Center

Department of Primary and Community Care

Postbus 9101, 6500 HB Nijmegen

The Netherlands

Email: Irene.gottgens@radboudumc.nl
